# Estimation of genetic parameters for superovulatory response traits in Japanese Black cows

**DOI:** 10.1093/jas/skab265

**Published:** 2021-10-07

**Authors:** Atsushi Zoda, Manami Urakawa, Yoshio Oono, Shinichiro Ogawa, Masahiro Satoh

**Affiliations:** 1 Research and Development Group, Zen-noh Embryo Transfer Center, Kamishihoro, Hokkaido, 080-1407, Japan; 2 Graduate School of Agricultural Science, Tohoku University, Sendai, Miyagi, 980-8572, Japan

**Keywords:** breeding value, embryo production, genetic parameter estimate, Japanese Black, superovulatory response

## Abstract

The aim of this study was to estimate genetic parameters for superovulatory response traits in order to explore the possibility of genetic improvement in Japanese Black cows. We analyzed 19 155 records of the total number of embryos and oocytes (TNE) and the number of good embryos (NGE) collected from 1532 donor cows between 2008 and 2018. A two-trait repeatability animal model analysis was performed for both. Because records of TNE and NGE did not follow a normal distribution, the records were analyzed following no, logarithmic, or Anscombe transformation. Without transformation, the heritability estimates were 0.26 for TNE and 0.17 for NGE. With logarithmic transformation, they were 0.22 for TNE and 0.18 for NGE. With Anscombe transformation, they were 0.26 for TNE and 0.18 for NGE. All analyses gave similar genetic correlations between TNE and NGE, ranging from 0.60 to 0.71. Spearman’s rank correlation coefficient between breeding values of cows with more than 10 records was ≥0.95 with both transformations. Thus, the genetic improvement of TNE and NGE of donor cows could be possible in Japanese Black cattle.

## Introduction

Japanese Black cattle are a Wagyu breed famous for their excellent meat quality (Gotoh et al., 2014). For Japanese Black cattle in Japan, embryo transfer is widely used in the production of commercial animals as well as breeding stocks. The value of Japanese Black calves is much higher than the other beef breeds, and therefore, it is profitable for dairy farmers to transfer embryos from Japanese Black cows to dairy cattle recipients. In Japan, in 2014, Japanese Black embryos were transferred into about 100 000 Holstein cows, and 42 000 calves were born, or 8% of the total number of Japanese Black calves born in 2014. Thus, embryo transfer plays an important role in efficient Wagyu production (Agriculture and Livestock Industry Promotion Organization, 2019). Reproductive techniques such as multiple ovulation, embryo transfer, and ovum pickup are widely used in dairy cattle production (Jaton et al., 2016); around the world, 470 000 bovine embryos were produced *in vivo* in 2018 (International Embryo Transfer Society [IETS], 2018). The number of embryos and oocytes obtained from donor dairy cows per flash is used as an indicator of the response to superovulation treatment (e.g., Jaton et al., 2016; Parker Gaddis et al., 2017). Large differences in numbers produced *in vivo* among cows have been reported (e.g., Kafi and McGowan, 1997; Kanitz et al., 2002; Mapletoft et al., 2002). The heritabilities of *in vivo* embryo production traits have been estimated for Holstein (Jaton et al., 2016; Parker Gaddis et al., 2017), Belgian Blue (Michaux et al., 2002), and Nellore (Zebu) cattle breeds (Peixoto et al., 2004), but not so far in Japanese Black cattle. This study estimated genetic parameters of superovulatory response traits in Japanese Black cows to assess the possibility of genetic improvement for embryo production.

## Materials and Methods

Animal Care and Use Committee approval was not needed because information was obtained from preexisting databases.

### Phenotypic measurements

The total number of embryos and oocytes recovered (TNE) and the number of good embryos (NGE) per flush were recorded from 20 257 superovulation treatments of 1532 Japanese Black donor cows between 2008 and 2018 at the Zennoh Embryo Transfer center, Hokkaido, Japan. TNE was defined as the sum of the number of embryos and unfertilized oocytes collected in a single flush; NGE was the number of embryos morphologically classified as grade 1 according to the International Embryo Transfer Society criteria (Robertson and Nelson, 1998). We analyzed 19 155 records of those with TNE ≥ 1.

After their first calving, all cows received superovulation treatment every ≥70 d (81.4 ± 27.2 d in average). As a basic program, first, a total of 20 AU of FSH (Antrin R-10, Kyoritsu Seiyaku Corp., Tokyo, Japan) was administered intramuscularly in the neck twice a day for 3 d. At the fifth treatment, PGF2α (cloprostenol 0.225 mg/cow, Darmajin, Kyoritsu Seiyaku Corp., Tokyo, Japan) was administered. The day after estrus was observed, cows were inseminated artificially. One week later, embryos were collected by washing of the uterine horns. Because embryo production performance from the same cow declines with the number of collections performed (Donaldson and Perry, 1983), the dosage of FSH was adjusted.

**Figure 1.**
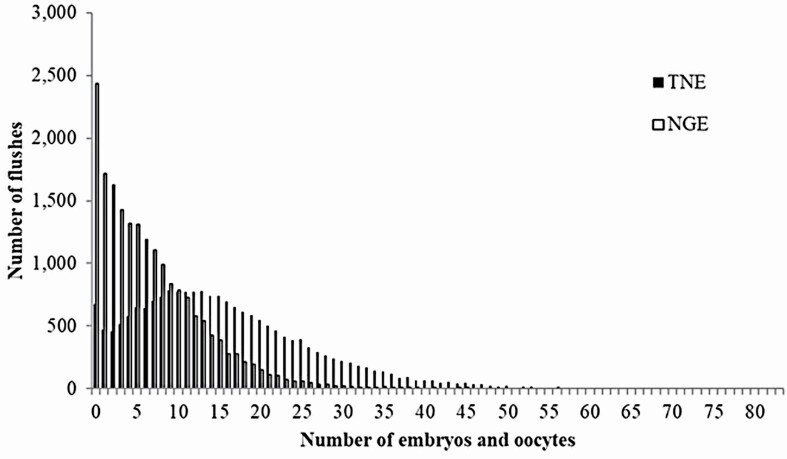
Histogram illustrating distributions of records of the total number of embryos and oocytes (TNE) and the number of good embryos (NGE).


As the distributions of TNE and NGE differed from normal distribution (Figure 1), logarithmic and Anscombe (ans) transformations were used (Jaton et al., 2016; Parker Gaddis et al., 2017) as follows:

$$\begin{array}{l} TNE_{log}\;=\;ln\;(TNE) \end{array}$$

$$\begin{array}{l} TNE_{ans}=2\times \sqrt{TNE\;+\;\frac{3}{8},} \end{array}$$

$$\begin{array}{l} NGE_{\log}=ln\left(NGE\;+\;1\right), \end{array}$$

$$\begin{array}{l} NGE_{ans}=2\times \sqrt{NGE\;+\;\frac{3}{8}.} \end{array}$$

### Statistical analysis

The following two-trait repeatability animal model was used to estimate genetic parameters:


$$\begin{array}{l} \left[\begin{array}{l} y_{1}\\ y_{2} \end{array}\right]=\;\left[\begin{array}{cc} X_{1} & 0\\ 0 & X_{2} \end{array}\right]\left[\begin{array}{l} b_{1}\\ b_{2} \end{array}\right]+\;\left[\begin{array}{cc} Z_{1} & 0\\ 0 & Z_{2} \end{array}\right]\left[\begin{array}{l} a_{1}\\ a_{2} \end{array}\right]+\;\left[\begin{array}{cc} W_{1} & 0\\ 0 & W_{2} \end{array}\right]\left[\begin{array}{l} pe_{1}\\ pe_{2} \end{array}\right]+\;\left[\begin{array}{l} e_{1}\\ e_{2} \end{array}\right] \end{array}$$

$$\begin{array}{ll} & E\left[\begin{array}{l} a_{1}\\ a_{2}\\ \begin{array}{l} pe_{1}\\ pe_{2}\\ \begin{array}{l} e_{1}\\ e_{2} \end{array} \end{array} \end{array}\right]=\;\left[\begin{array}{l} 0\\ 0\\ \begin{array}{l} 0\\ 0\\ \begin{array}{l} 0\\ 0 \end{array} \end{array} \end{array}\right],\;and\;var\left[\begin{array}{l} a_{1}\\ a_{2}\\ \begin{array}{l} pe_{1}\\ pe_{2}\\ \begin{array}{l} e_{1}\\ e_{2} \end{array} \end{array} \end{array}\right]\\ & =\;\left[\begin{array}{cccccc} \sigma_{a1}^{2}A & \sigma_{a12}A & 0 & 0 & 0 & 0\\ \sigma_{a12}A & \sigma_{a2}^{2}A & 0 & 0 & 0 & 0\\ 0 & 0 & \sigma_{pe1}^{2}I & \sigma_{pe12}I & 0 & 0\\ 0 & 0 & \sigma_{pe12}I & \sigma_{pe2}^{2}I & 0 & 0\\ 0 & 0 & 0 & 0 & \sigma_{e1}^{2}I & \sigma_{e12}I\\ 0 & 0 & 0 & 0 & \sigma_{e12}I & \sigma_{e2}^{2}I \end{array}\right] \end{array}$$

where y_i_ is the vector of phenotypic records (*i* = 1 for TNE, *i* = 2 for NGE); $b_{i}$, $a_{i}$, $pe_{i}$, and *e*_*i*_ are the vectors of fixed effects (year of superovulation, month of superovulation, type of superovulation program, technician, and linear and quadratic covariates of age in months at superovulation), breeding values of cows, permanent environmental effects of cows, and errors; $X_{i}$, $Z_{i}$, and $W_{i}$ are the design matrices relating $y_{i}$ to $b_{i}$, $a_{i}$, and $pe_{i}$, respectively; $\sigma_{ai}^{2}$ is the additive genetic variance of trait *i*, $\sigma_{a12}$ is the additive genetic covariance between traits, $\sigma_{pei}^{2}$ is the permanent environmental variance of trait *i*; $\sigma_{pe12}$ is the permanent environmental covariance between traits; $\sigma_{ei}^{2}$ is the error variance for trait *i*; $\sigma_{e12}$ is the error covariance between traits; *A* is the additive genetic relationship matrix constructed from pedigree information of 3,521 individuals; and *I* is the identity matrix. As preliminary check, we confirmed the significance of all the fixed effects in the model (*P* < 0.0001) by least squares analysis. The effect of service sire was not included because previous studies reported the impact of service sire was estimated to be negligible (König et al., 2007; Jaton et al., 2016). We did not include the effect of the stage of lactation because our donors basically do not calve again after first calving and the effect of the number of flushes because it could be highly confounded with the age in this study.

Variance components were estimated in AIREMLF90 software (Misztal et al., 2002) with the default convergence criterion (10^−12^). The SEs of the estimated heritability, repeatability, and genetic correlation were calculated by using the se_cover_function option (Houle and Meyer, 2015).

Reliabilities of estimated breeding values (EBVs) for cow *i* ($r_{i}^{2}$) were calculated as follows:


$$\begin{array}{l} r_{i}^{2}=1-\frac{PEV_{i}}{\left(1+F_{i}\right){\hat{\sigma }}_{a}^{2}} \end{array}$$

where $F_{i}$ is the inbreeding coefficient of cow *i*, and $PEV_{i}$ is the prediction error variance of EBV of cow *i*.

## Results and Discussion

### Descriptive statistics of phenotypic records

**Figure 2.**
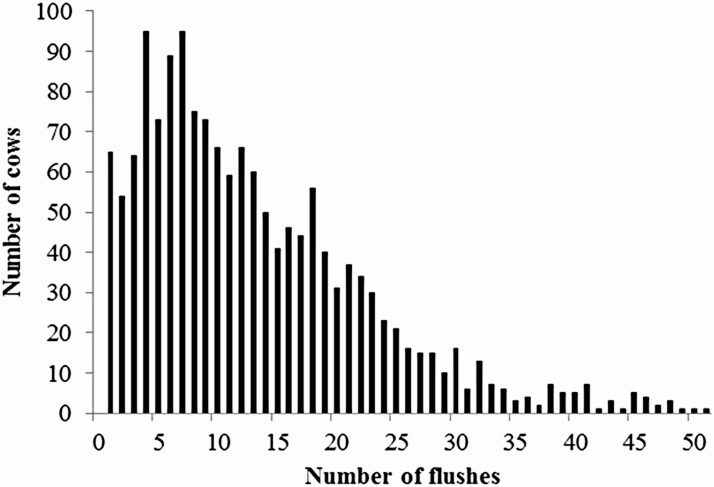
Histogram of the number of flushes per donor cow.

Figure 1 shows the histogram illustrating the distributions of records of TNE and NGE. Figure 2 shows the number of flushes per donor. Basic statistics of superovulatory response traits are listed in Table 1. The mean TNE in this study (16.50) was higher than those reported in previous studies of Holstein (6.67, Asada and Terawaki, 2002; 9.21, Jaton et al., 2016; 9.27, Cornelissen et al., 2017), Belgian Blue (6.68, Michaux et al., 2002), and Nellore (10.27, Peixoto et al., 2004). The mean NGE (6.73) was higher than that of Holstein (5.11, Gaddis et al., 2017). Because beef breeds could be more responsive to superovulation treatments than dairy breeds, beef cattle might have the ability to produce more embryos per flush (Mikkola et al., 2020). Steinhauser et al. (2018) found that Wagyu breeds responded better to superovulation treatments than other *Bos taurus* and *Bos indicus* breeds, but they did not describe the treatment conditions. Yokoo et al. (2016) reported that Japanese Black cows responded better than Japanese Shorthorn cows. Therefore, the Japanese Black cattle could have the potential for high response to superovulation treatments, as our results suggest.

**Table 1.** Basic statistics for the total number of embryos and oocytes (TNE) and the number of good embryos (NGE)

Trait	No. of records	No. of cows	Mean	SD	Minimum	Maximum
TNE	19 155	1 532	16.50	10.66	1	84
NGE			6.73	6.12	0	56

**Figure 3.**
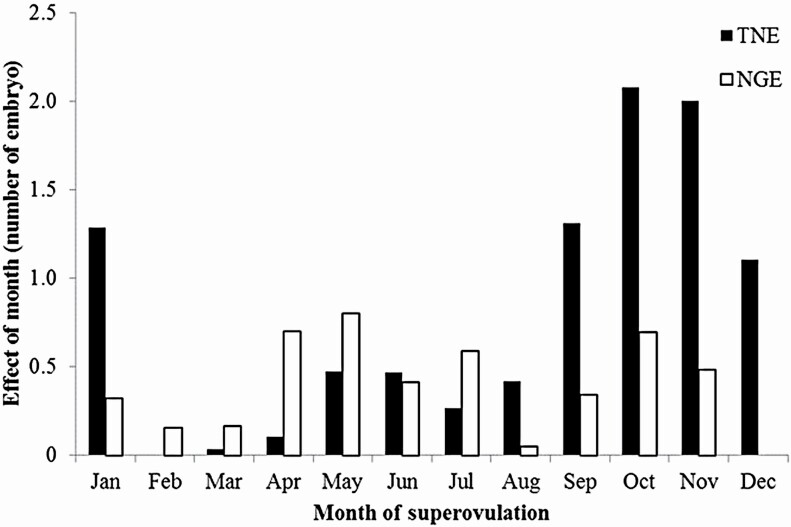
Effects of month of superovulation on the total number of embryos and oocytes (TNE) and the number of good embryos (NGE).

Figure 3 shows the effects of month of superovulation obtained from the analysis using the untransformed data. The difference in TNE between the maximum and minimum values of the estimated effects was approximately 2 embryos. NGE tended to be lower in winter. Heat stress could affect the estrus cycle and sign in Japanese Black and Holstein cows (Sakatani et al., 2012), and cold stress could also affect fertility and reproductive performance in Japanese Black cows (Nabenishi and Yamazaki, 2017; Kino et al., 2019). Because the data were collected in subarctic Hokkaido, cold stress might be dominant in this Japanese Black population.

**Figure 4.**
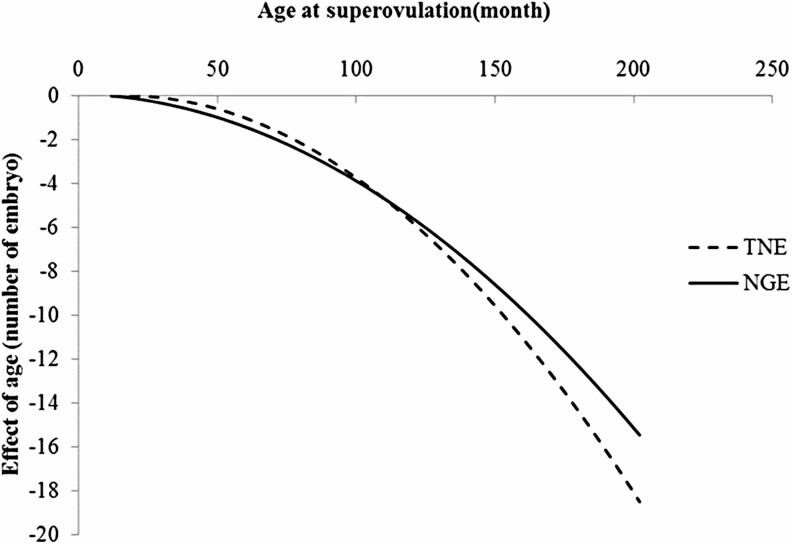
Effects of age at superovulation for the total number of embryos and oocytes (TNE) and the number of good embryos (NGE).

Figure 4 shows the effect of age at superovulation obtained from the analysis using the untransformed data. The effects would be very small (Mikkola et al., 2020), but the effect of the number of flushes previously experienced is still unclear. Our results indicate that the response decreased with increasing age of the cow, perhaps because responsiveness to FSH would decrease by undergoing the superovulation over and over again. In our data, mean age (mo) of superovulation was 66. Peixoto et al. (2004) using 1,036 superovulation records of 475 Nellore females, whose ages ranged from 2.2 to 20.5 yr old at the time of superovulatory treatment, and 3%, 25%, 30%, 23%, and 19% of them were <3, 3–5.9, 6–8.9, 9–11.9, and >12 yr old, respectively. Therefore, the mean age of superovulation in Peixoto et al. (2004) could be older than our data. Higher means of superovulatory response traits in our study might be due to younger age of superovulation than previous studies.

### Estimated genetic parameters

Genetic parameter estimates of TNE and NGE and their standard errors (SE) are listed in Table 2. Without transformation, the estimated heritability was 0.26 for TNE and 0.17 for NGE. The heritability of NGE was also lower in Holstein (Parker Gaddis et al., 2017). The heritability of TNE estimated here was similar to previous estimates in Holstein (Jaton et al., 2016; Parker Gaddis et al., 2017) and Belgian Blue (Michaux et al., 2002). The estimated repeatability was 0.37 for TNE and 0.26 for NGE, both similar to previous estimates in Holstein (Jaton et al., 2016; Parker Gaddis et al., 2017).

**Table 2.** Genetic parameter estimates ± SE of the total number of embryos and oocytes (TNE) and the number of good embryos (NGE) using untransformed, log-transformed, and Anscombe-transformed data

Transformation	Trait	$\sigma_{a}^{2}$	$\sigma_{pe}^{2}$	$\sigma_{e}^{2}$	$h^{2}$	rep^2^	r_g_
Untransformed	TNE	29.18 ± 5.02	11.74 ± 3.53	69.65 ± 0.74	0.26 ± 0.04	0.37 ± 0.01	0.71 ± 0.08
	NGE	6.11 ± 1.27	3.49 ± 0.92	26.85 ± 0.29	0.17 ± 0.03	0.26 ± 0.01	
Logarithm	TNE	0.16 ± 0.03	0.07 ± 0.02	0.49 ± 0.01	0.22 ± 0.04	0.32 ± 0.01	0.60 ± 0.09
	NGE	0.15 ± 0.03	0.06 ± 0.02	0.63 ± 0.01	0.18 ± 0.03	0.25 ± 0.01	
Anscombe	TNE	1.81 ± 0.31	0.73 ± 0.22	4.39 ± 0.05	0.26 ± 0.04	0.37 ± 0.01	0.65 ± 0.08
	NGE	0.98 ± 0.19	0.43 ± 0.13	3.90 ± 0.04	0.18 ± 0.03	0.27 ± 0.01	

$\sigma_{a}^{2}$ , additive genetic variance$;\;\sigma_{pe}^{2}$, permanent environmental variance; $\sigma_{e}^{2}$, error variance; $h^{2}$, heritability; rep^2^, repeatability; *r*_g_, genetic correlation.

The estimated genetic correlation between TNE and NGE was 0.71 without transformation. Parker Gaddis et al. (2017) estimated a correlation of almost 1 in Holstein. High positive genetic correlations (0.74–0.97) between TNE and the number of transferable embryos have been estimated in Holstein (König et al., 2007; Jaton et al., 2016) and Belgian Blue (Michaux et al., 2002). Our estimated genetic correlation was slightly lower than in previous studies. Cattle breeds might affect the genetic correlation between TNE and NGE.

### Effect of transformation of phenotypic records

With logarithmic transformation, heritability estimates were 0.22 for TNE and 0.18 for NGE, and repeatability estimates were 0.32 and 0.25 (Table 2). With Anscombe transformation, heritability estimates were 0.26 for TNE and 0.18 for NGE and repeatability estimates were 0.37 and 0.27. Estimates of genetic correlation between TNE and NGE were 0.60 with logarithmic transformation and 0.65 with Anscombe transformation. Thus, the estimates of heritability, repeatability, and genetic correlation differed little between untransformed and transformed data.

### Reliabilities of estimated breeding values

**Figure 5.**
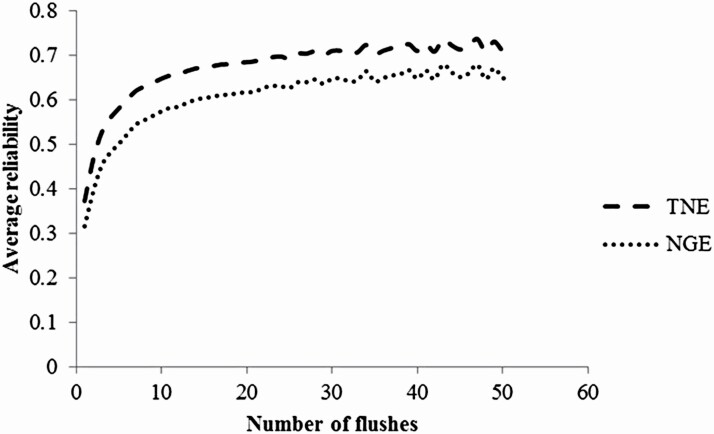
Relationships between the number of flushes per donor cow and the average reliability of estimated breeding values for the total number of embryos and oocytes (TNE) and the number of good embryos (NGE).

Figure 5 shows the relationship between the number of flushes and the mean reliability of estimated breeding values of cows by two-trait repeatability animal model analysis using untransformed data. The mean reliability initially increased rapidly with the number of records, but then increased more slowly beyond 10 flushes. Spearman’s correlation coefficients among the EBVs of cows (*n* = 805) with ≥10 records are listed in Table 3. Values of both TNE and NGE were always ≥0.95. The values of the rank correlation coefficients were almost the same (≥0.95) even when all cows with records were included. This indicates that the difference in the genetic ability of cows selected by using untransformed and transformed records was small.

**Table 3.** Spearman’s rank correlation coefficients of estimated breeding values of cows with 10 or more superovulation records

	Untransformed	Logarithm	Anscombe
Untransformed	–	0.97	0.99
Logarithm	0.95	–	0.99
Anscombe	0.98	0.99	–

Above the diagonal: the total number of embryos and oocytes; below the diagonal: the number of good embryos.

## General Discussion

In this study, genetic parameters of TNE and NGE were estimated as traits relating to response to superovulation treatments, the first such report in Japanese Black cattle. The heritabilities of both were moderate and the genetic correlation between them was positive and high. Both had higher heritabilities than other female reproductive traits such as calving interval, non-return rate, and conception rate (Oyama et al., 2002; VanRaden et al., 2004; Inoue et al., 2020; Ogawa and Satoh, 2021), and the accuracy of selection for TNE and NGE could be higher than those for other representative female reproductive traits. Jaton et al. (2016) and Parker Gaddis et al. (2017) reported that genetic improvement by selection for superovulatory response in Holstein cows was possible. Our results indicate that it is possible also in Japanese Black.

The data analyzed here differed from those in previous studies in terms of the greater number of records. The average number of repeated records per cow in previous studies was 1 to 3 (Michaux et al., 2002; Peixoto et al., 2004; Jaton et al., 2016; Cornelissen et al., 2017; Parker Gaddis et al., 2017), whereas that here was 12.5. Furthermore, the total number of records analyzed here was larger than in previous studies of beef cattle (Michaux et al., 2002) and dual-purpose breeds (Peixoto et al., 2004). The reliabilities of EBV of both traits were around 0.6 when the number of repeated records was 10, but it increased only slowly beyond this (Figure 5). In our population, superovulation records can be collected about once every 3 mo. As the mean age at first calving is about 24 mo in Japanese Black cows, we can obtain 10 repeated records of TNE and NGE at 5 yr of age. Furthermore, the genetic correlation between them was high and positive. There were little differences in the estimated genetic parameters with and without transformation and the values of rank correlation coefficients of EBVs were very high. We concluded that the two-trait animal model analysis with untransformed data would be preferable for predicting breeding values for TNE and NGE because of the ease of handling data.

The superovulatory response decreased with age (Figure 4). The proportion of the number of high-quality embryos also tended to decrease with age (results not shown). It would be possible that some cows have good response for many times of flushes, whereas others quickly become unresponsive to superovulation. Female reproductive traits in cattle, such as calving interval (Panetto et al., 2012; Ogawa and Satoh, 2021), could be analyzed using a random regression model, in terms of gene-environment (age) interaction. Such kinds of analysis would be needed in the future. On the other hand, improving representative carcass traits, including degree of marbling, is economically most important in Japanese Black cattle (Sasaki et al., 2006; Oyama., 2011). Hirayama et al. (2019) investigated superovulatory responses among lines of Japanese Black cattle and reported that strains with better superovulatory responses had phenotypically superior growth. It is important to select cows with better TNE and NGE without expense of other economically important traits such as body weight, carcass weight, and meat quality. Hence, research to investigate the genetic relationships between carcass and embryo production traits in Japanese Black cattle will be needed.

## Conclusion

We estimated genetic parameters for TNE and NGE in Japanese Black cattle. Estimated heritabilities were moderate and estimated genetic correlation was high. The estimates of heritability and genetic correlation differed little between untransformed and transformed data. The values of rank correlation coefficients of EBVs between with and without transformation were very high. These results suggest that genetic improvement of both by selection is possible in Japanese Black cows.

## Abbreviations

EBV: estimated breeding value
IETS: International Embryo Transfer Society
NGE: number of good embryos
TNE: total number of embryos and oocytes
